# MicroRNA-25 regulates chemoresistance-associated autophagy in breast cancer cells, a process modulated by the natural autophagy inducer isoliquiritigenin

**DOI:** 10.18632/oncotarget.2192

**Published:** 2014-07-09

**Authors:** Zhiyu Wang, Neng Wang, Pengxi Liu, Qianjun Chen, Honglin Situ, Ting Xie, Jianxing Zhang, Cheng Peng, Yi Lin, Jianping Chen

**Affiliations:** ^1^ Department of Mammary Disease, Guangdong Provincial Hospital of Chinese Medicine; ^2^ School of Chinese Medicine, The University of Hong Kong, Pokfulam, Hong Kong, China; ^3^ Department of Dermatology, Guangdong Provincial Hospital of Chinese Medicine, The Second Clinical Collage of Guangzhou University of Chinese Medicine; ^4^ Deapartment of Pharmacology, Chengdu University of Traditional Chinese Medicine

**Keywords:** Autophagy, Drug resistance, miRNA-25, ULK1, Isoliquiritigenin, Breast cancer

## Abstract

Recent findings have revealed that dysregulated miRNAs contribute significantly to autophagy and chemoresistance. Pharmacologically targeting autophagy-related miRNAs is a novel strategy to reverse drug resistance. Here, we report a novel function of isoliquiritigenin (ISL) as a natural inhibitor of autophagy-related miR-25 in killing drug-resistant breast cancer cells. ISL induced chemosensitization, cell cycle arrest and autophagy, but not apoptosis, in MCF-7/ADR cells. ISL also promoted the degradation of the ATP-binding cassette (ABC) protein ABCG2 primarily *via* the autophagy-lysosome pathway. More importantly, miRNA 3.0 array experiments identified miR-25 as the main target of ISL in triggering autophagy flux. A mechanistic study validated that miR-25 inhibition led to autophagic cell death by directly increasing ULK1 expression, an early regulator in the autophagy induction phase. miR-25 overexpression was demonstrated to block ISL-induced autophagy and chemosensitization. Subsequent *in vivo* experiments showed that ISL had chemosensitizing potency, as revealed by an increase in LC3-II staining, the downregulation of ABCG2, a reduction in miR-25 expression and the activation of the miR-25 target ULK1. Overall, our results not only indicate that ISL acts as a natural autophagy inducer to increase breast cancer chemosensitivity, but also reveal that miR-25 functions as a novel regulator of autophagy by targeting ULK1.

## INTRODUCTION

Breast cancer is the most common malignancy and the second leading cause of cancer death in women worldwide. With the development of multidisciplinary treatments, the 5-year survival rate of breast cancer has greatly been improved. However, there are still 500,000 breast cancer deaths per year worldwide [1]. Approximately 40% of all breast cancer patients experience a recurrence, of which 10–20% are local and 60–70% are distant metastases [2]. Drug resistance is considered a major factor influencing breast cancer clinical outcomes. However, current progress in finding a potent, selective agent to overcome cancer drug resistance has been slow and challenging [3]. How to effectively promote breast cancer chemosensitivity has become an urgent issue worldwide.

The overexpression of ATP-binding cassette (ABC) transporters is highly correlated with drug resistance [4]. However, ABC-targeting strategies have not been as effective as expected due to the involvement of various other mechanisms, such as alterations in drug metabolism, mutations in drug targets and changes in the tumor microenvironment [5]. More recently, autophagy induction has emerged as a novel strategy to overcome drug resistance during cancer therapy [6]. Meschini *et al.* found that the natural compound vocamine led to autophagic cell death in doxorubicin-resistant osteosarcoma cells, which was accompanied by a decrease in ABCB1 expression [7]. Another study reported that autophagy induction in apoptosis-deficient H460 lung cancer cells resulted in an enhanced efficacy of radiation therapy *in vitro* and *in vivo* [8]. Furthermore, it was found that the constitutive expression of ABCB1 in hepatocellular cancer cells was positively linked to the overexpression of Bcl2 and mTOR, rendering these cells resistant to autophagy [9]. However, enhanced autophagy was usually observed in advanced stages of tumorigenesis, and some studies have claimed that autophagy inhibition increased cancer chemosensitivity to cytotoxic drugs [10-14]. Therefore, autophagy can be considered a double-edged sword in cancer development, and autophagy modulation has become a novel strategy to overcome cancer drug resistance.

In the past decade, genetic screens in yeast have identified a large family of core autophagy-related genes (ATG), such as Atg1, Atg4, LC3/Atg8 and BECN1 [15]. There are additional contributions to autophagy regulation by a variety of upstream signaling pathways, including the phosphatidylinositol 3-kinase (PI3K), AMP-activated protein kinase (AMPK) and mammalian target of rapamycin complex 1 (mTORC1) pathways [16]. Recently, a group of endogenous noncoding miRNAs have been thoroughly investigated in autophagy modulation [17]. miRNAs are endogenous ~22 nucleotide RNAs that suppress gene expression *via* messenger RNA (mRNA) cleavage and/or translational repression. There is accumulating evidence that miRNAs play critical roles in a broad range of biological processes, including proliferation, differentiation, angiogenesis and stress response, linking them to a variety of human diseases, including cancer [18, 19]. Because a single miRNA can simultaneously regulate a multitude of targets and biological networks, increasing attention is being focused on developing miRNA-based strategies for cancer therapy. A series of miRNAs have been implicated in cancer patient survival and the modification of anticancer strategies. However, discoveries about the roles of miRNAs in mediating autophagy and drug resistance are currently limited. At present, only a small subset of miRNAs, including miR-30a, miR-23b and miR-199a-5p, have been confirmed to regulate cancer chemosensitivity *via* autophagy-related processes [20, 21]. Nonetheless, it is worthwhile to develop pharmacological agents targeting these dysregulated miRNAs to restore drug sensitivity [22].

Candidate drugs for reversing drug resistance should ideally be selective, potent and relatively nontoxic [3]. Because natural extracts are usually low in toxicity and are well-tolerated in the human body, increasing attention has been paid to discovering chemosensitizing agents from natural sources. Isoliquiritigenin (ISL) is a natural flavonoid isolated from the root of licorice (*Glycyrrhiza uralensis*), which is commonly used for culinary purposes in Western countries but as a medicinal herb in China. Previous research has demonstrated that ISL possesses various biologic properties, such as anti-inflammatory, anti-oxidant, anti-platelet aggregation, vasorelaxant and estrogenic effects [23]. A number of studies have reported significant antitumor activities of ISL, including apoptosis induction, cell cycle arrest, migration inhibition and oxidative stress triggering [24-28]. Some studies have indicated that ISL mediates chemopreventive activities, including suppressing 7,12-dimethylbenz[a]anthracene (DMBA)-induced mouse skin carcinogenesis and inhibiting carcinogen-induced lesion formation in a mouse mammary organ culture assay [29, 30]. Very recently, we demonstrated that ISL inhibited breast cancer neoangiogenesis by specifically docking into the ATP domain of VEGFR-2 kinase [31]. These findings suggest that ISL is a multifunctional compound with anticancer properties. However, there has been little research into the chemosensitizing effects of ISL and its association with autophagy and miRNAs.

In the present study, we revealed a novel activity for ISL as a potent autophagy inducer to chemosensitize drug-resistant breast cancer cells. We showed that ISL induced proliferation inhibition, cell cycle arrest and autophagy, but not apoptosis, in drug-resistant breast cancer cells. ISL was found to promote the influx of chemotherapeutic agents by promoting ABCG2 degradation through the autophagy-lysosome pathway. An miRNA 3.0 array analysis and a functional study further revealed that miR-25 is the primary target of ISL and a novel autophagy-related miRNA. A mechanistic study validated that miR-25 inhibition led to autophagic cell death by directly increasing the expression of ULK1, an early regulator in the autophagy induction phase. Notably, ISL-induced autophagy was demonstrated to be dependent on the function of miR-25 in both *in vitro* and *in vivo* assays. Our study not only demonstrated that ISL is a natural autophagy inducer to increase breast cancer chemosensitivity but also elucidated the role of miR-25 as a novel regulator of autophagy modulation by targeting ULK1.

## RESULTS

### ISL chemosensitizes drug-resistant breast cancer cells

**Figure 1:**
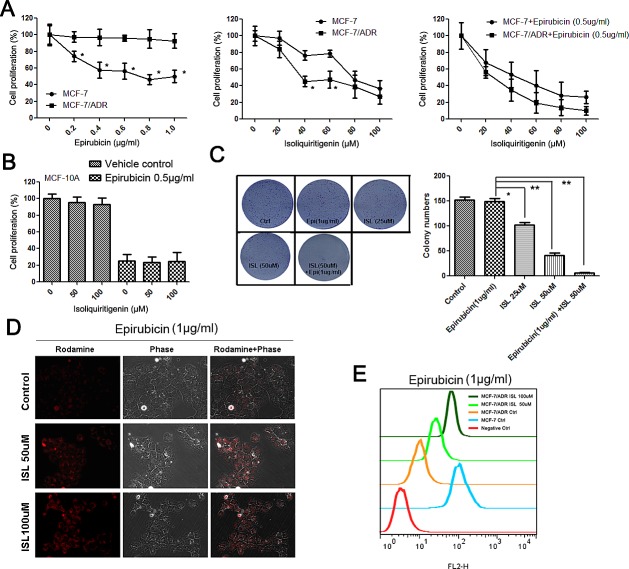
ISL chemosensitizes MCF-7/ADR breast cancer cells (A) ISL and epirubicin exerted synergistic effects to inhibit the proliferation of MCF-7/ADR cells after 24 h of treatment (the values represent the means ± SD, n=6, *P<0.05 MCF-7/ADR vs. MCF-7); (B) ISL produced minimal inhibitory effects on the proliferation of the MCF-10A normal human mammary epithelial cell line and did not exacerbate the cytotoxic effects of epirubicin after 24 h of treatment (the values represent the means ± SD, n=6); (C) ISL (alone or in combination with epirubicin) significantly inhibited the colony-forming ability of MCF-7/ADR cells (the values represent the means ± SD, n=3, *P<0.05, **P<0.01); (D) ISL treatment dose-dependently increased the intracellular concentration of epirubicin; (E) flow cytometry revealed that ISL promoted the influx of epirubicin into MCF-7/ADR cells.

To determine whether ISL had chemosensitizing effects on drug-resistant breast cancer cells, we tested the synergistic effects of ISL and the chemotherapeutic drug epirubicin, which is usually administered as the first-line chemotherapy for breast cancer. As shown in Figure 1A, epirubicin induced limited proliferation inhibition in drug-resistant breast cancer MCF-7/ADR cells compared with its effects on MCF-7 cells. However, ISL had significant inhibitory effects on the proliferation of MCF-7/ADR cells and interacted synergistically with epirubicin to induce cell death. In contrast, ISL had limited inhibitory effects on the proliferation of normal human mammary epithelial MCF-10A cells and did not increase the cytotoxic effects of epirubicin, indicating that ISL may be a safe chemosensitizing agent (Figure 1B).

To determine the long-term inhibitory effects of ISL on the proliferation of MCF-7/ADR cells, we performed a colony formation assay. The results showed that ISL alone dose-dependently inhibited the colony growth of MCF-7/ADR and significantly limited the colony number when combined with epirubicin (Figure 1C). The intracellular concentration of epirubicin was also greatly elevated in ISL-treated cancer cells, as revealed using fluorescence microscopy (Figure 1D) and flow cytometry (Figure 1E). These results indicated that ISL inhibited the drug-resistant property of MCF-7/ADR cells and promoted epirubicin influx.

### ISL induces autophagy, but not apoptosis, in drug-resistant breast cancer cells

**Figure 2:**
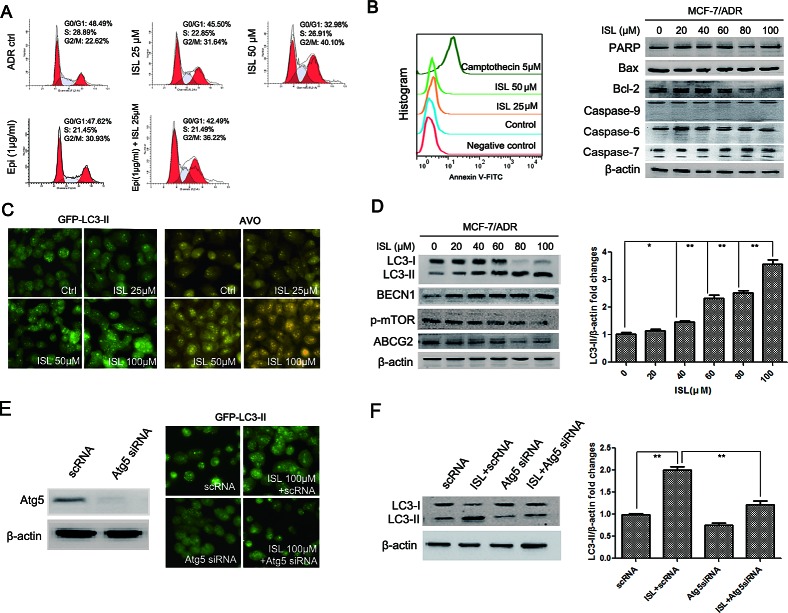
ISL induces autophagy in MCF-7/ADR cells (A) ISL dose-dependently arrested the MCF-7/ADR cell cycle at the G2/M checkpoint after 24 h of treatment. Epirubicin cooperated with ISL to inhibit mitosis; (B) Annexin V-FITC staining and western blotting showed that ISL induced minimal apoptosis in MCF-7/ADR cells at 24 h but downregulated Bcl-2 expression; (C) ISL triggered autophagy in MCF-7/ADR cells after 24 h of treatment, indicated by increased LC3-II puncta and AVO formation; (D) western blotting revealed that ISL activated autophagy flux in ADR cells, indicated by increased levels of LC3-II and BECN1, decreased expression of p-mTOR and inhibited expression of ABCG2; (E) the autophagy-inducing effect of ISL was blocked by Atg5 siRNA, as indicated by decreased LC3-II puncta; (F) western blotting revealed that Atg5 siRNA treatment inhibited the ISL-induced increase in LC3-II expression.

To elucidate the mechanism underlying the ability of ISL to chemosensitize MCF-7/ADR cells, we first examined the potential influence of ISL on cell cycle progression. The results showed that ISL alone dose-dependently arrested the cell cycle at the G2/M checkpoint. Additionally, when cells were treated with a combination of ISL and epirubicin, the G2/M population increased to 36.22% after 24 h, which was significantly higher than the G2/M proportion of 31.64% in the cells treated with ISL alone (Figure 2A). These results revealed that ISL interacted synergistically with epirubicin to inhibit cell mitosis. We then investigated whether ISL could induce apoptosis in drug-resistant breast cancer cells. A flow cytometry assay revealed the presence of few Annexin V-positive cells following ISL addition compared with the positive control, camptothecin. A western blotting assay also indicated that ISL had minimal effects on apoptosis markers, including cleaved PARP (poly ADP-ribose polymerase), caspase-6, caspase-7 and caspase-9. However, the protein expression of Bcl-2 was greatly downregulated after ISL treatment (Figure 2B). Because Bcl-2 is reported to be an important regulator of autophagy by binding to BECN1 [32], we examined whether ISL could trigger autophagy in MCF-7/ADR cells.

As shown in Figure 2C, ISL treatment led to a significant dose-dependent increase in the autophagy activity of ADR cells, indicated by increased fluorescent intensity of GFP-LC3-II puncta. Acridine orange (AO) staining revealed that after ISL treatment, the intracellular formation of acidic vesicular organelles (AVOs) was greatly increased, indicating enhanced activity of autophagosomes and lysosomes. Western blotting results showed that ISL significantly increased the expression of LC3-II, which was accompanied by the upregulation of BECN1 and downregulation of p-mTOR and ABCG2 (Figure 2D). To confirm the autophagy-inducing effects of ISL, we transfected MCF-7/ADR cells with siRNA targeting Atg5, a critical protein responsible for autophagosome formation [33]. As shown in Figure 2E, Atg5 siRNA significantly blocked the increases in GFP-LC3-II puncta and AVO formation induced by ISL treatment. The ISL-induced upregulation of LC3-II was also inhibited by Atg5 siRNA, further validating the autophagy-inducing effects of ISL (Figure 2F). Taken together, these results revealed that ISL triggered the intracellular autophagy, which was accompanied by the downregulation of ABCG2. Because ABCG2 is the primary membrane transporter responsible for drug resistance in breast cancer, our findings implied that the chemosensitizing effects of ISL might be closely correlated with ABCG2 reduction *via* the autophagy pathway.

### ISL induces ABCG2 degradation *via* the autophagy-lysosome pathway

**Figure 3:**
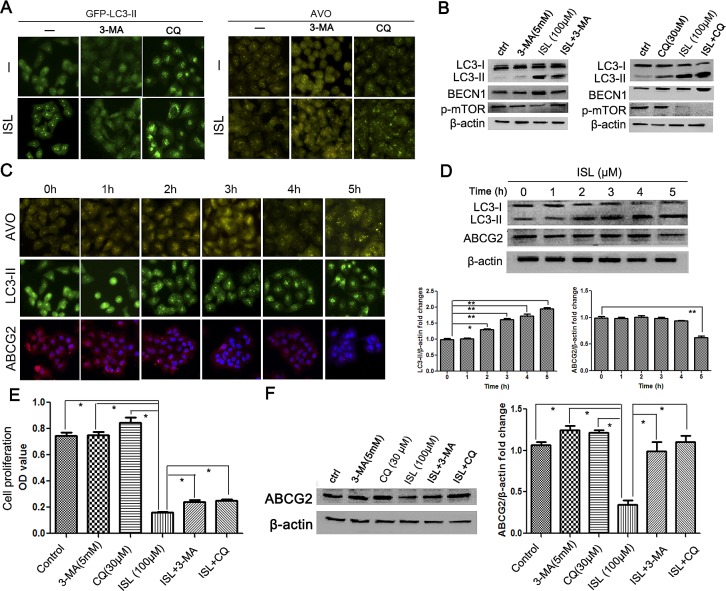
ISL inhibits ABCG2 expression via the autophagy-lysosome pathway (A–B) Treatment with the autophagy inhibitor 3-MA abolished the autophagy-inducing effects of ISL after 24 h of treatment, indicated by decreases in LC3-II expression and AVO formation, whereas adding the lysosome inhibitor CQ enhanced LC3-II accumulation and AVO formation in ADR cells after 24 h, indicating that the ISL-induced enhancement of autophagy markers was due to autophagy induction rather than the inhibition of autophagosome maturation; these results were further validated by a western blotting analysis; (C–D) time point-based immunofluorescence detection was performed to examine the correlation between autophagy induction and ABCG2 inhibition. The results showed that AVO formation and LC3-II accumulation occurred as early as 2 h after ISL treatment, whereas decreased ABCG2 expression was observed at the 5^th^ hour, indicating that autophagy induction occurred prior to the downregulation of the drug resistance marker ABCG2; the results were validated by western blotting; (E–F) 24 h of treatment with the autophagy inhibitors 3-MA and CQ restored the cell viability and ABCG2 expression inhibited by ISL, indicating that ISL may induce cell death primarily by the autophagy pathway (the values represent the means ± SD, n=3, *P<0.05, **P<0.01).

To confirm that the enhancement of autophagy markers by ISL was due to the induction of autophagy rather than the blockage of autophagosome maturation, the lysosome inhibitor chloroquine (CQ) was added to the culture medium along with ISL. Both the LC3-II level and AVO formation in the cotreatment group were significantly higher than in the group treated with CQ alone (Figure 3A). Western blotting results demonstrated that ISL further induced LC3-II upregulation following CQ treatment (Figure 3B). ISL-induced autophagy was abolished by the autophagy inhibitor 3-methyladenine (3-MA). The addition of 3-MA led to the inhibition of both ISL-induced AVO formation and the upregulation of LC3-II and BECN1 (Figure 3A,B). Because 3-MA is well known as an autophagy inhibitor targeting the upstream PI3K signaling pathway [34], the results indicated that ISL functioned by inducing the early stage of autophagy flux rather than by inhibiting lysosome degradation.

Because the mature oligomeric ABCG2 is reported to be degraded primarily *via* the lysosome pathway [35], we therefore investigated whether the downregulation of ABCG2 induced by ISL was due to autophagy induction. We performed a time point-based fluorescence detection of AVOs and LC3-II and ABCG2 expression. The results showed that after ISL treatment for 2 h, MCF-7/ADR cells exhibited significantly elevated numbers of AVOs, which was accompanied by increased expression of LC3-II. However, the fluorescence intensity of ABCG2 was inhibited until the 5^th^ hour after ISL treatment, indicating that autophagy induction by ISL occurred prior to ABCG2 downregulation (Figure 3C). The expression level of ABCG2 also began to decrease at the 5^th^ hour, indicating that the autophagy-lysosome pathway may be the main mechanism underlying the ABCG2 reduction induced by ISL (Figure 3D).

To determine whether autophagy inhibition would reduce the death-inducing effects of ISL on drug-resistant breast cancer cells, we added 3-MA or CQ along with ISL to MCF-7/ADR cells and assessed cell proliferation. The results showed that 3-MA and CQ inhibited the proliferation of MCF-7/ADR cells to a limited extent. However, the cancer cell death induced by ISL was reduced to some degree by both 3-MA and CQ (Figure 3E). ISL also arrested the cell cycle at the G2/M checkpoint, an important factor in proliferation, which may explain why 3-MA and CQ could not fully abolish the inhibitory effects of ISL on proliferation. A western blotting assay revealed that 3-MA or CQ addition restored the ABCG2 expression that was downregulated by ISL (Figure 3F). These results indicated that ISL chemosensitized the drug-resistant breast cancer cells primarily *via* the autophagy-lysosome pathway.

### Identification of miR-25 as the main autophagy-related modulator targeting ULK-1 using ISL

**Figure 4:**
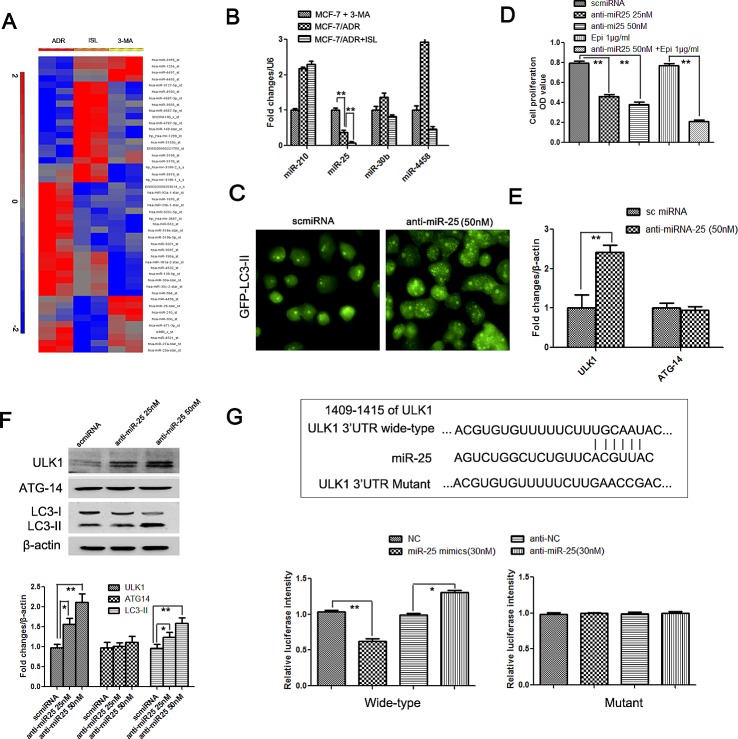
Identification of miR-25 as a novel regulator of ISL-induced autophagy, which functions by targeting ULK1 (A) An miRNA 3.0 array was used to detect miRNA expression differences between 3-MA-treated MCF-7/ADR cells, untreated MCF-7/ADR cells and ADR cells treated with ISL for 24 h. As a result, miR-210, miR-25, miR-30b and miR-4458 were found to exhibit expression patterns that were correlated with autophagy activity; (B) a qPCR analysis validated miR-25 as the primary regulator of autophagy, indicated by its gradually decreased expression in 3-MA-treated MCF-7/ADR, MCF-7/ADR and ISL-treated ADR cells (the values represent the means ± SD, n=3, **P<0.01); (C) miR-25 inhibitors were transfected into MCF-7/ADR cells. After 24 h, increases in GFP-LC3-II accumulation was observed, confirming that miR-25 independently induced autophagy flux; (D) miR-25 inhibitor treatment for 24 h resulted in decreased cell proliferation and significantly increased chemosensitivity to epirubicin, indicating that miR-25 may also be strongly connected to cancer chemosensitivity (the values represent the means ± SD, n=6,** *P*<0.01 vs. normal controls); (E–F) qPCR and western blotting results validated that miR-25 inhibition led to an increase in ULK1 but not in ATG-14, indicating that the direct target of miR-25 could be ULK1 (the values represent the means ± SD, n=3, *P<0.05, **P<0.01); (H) a luciferase reporter gene assay further confirmed that ULK1 transcription was inhibited by miR-25 treatment but enhanced by miR-25 inhibitors. However, when the predicted binding sites of miR-25 in the 3′UTR region of ULK1 were mutated, miR-25 mimics and inhibitors had little influence on the transcription of ULK1, indicating that miR-25 binds directly to the predicted sites in the 3′UTR region of ULK1 (the values represent the means ± SD, n=3, *P<0.05, **P<0.01).

To identify the miRNA target of ISL during autophagy induction, an miRNA 3.0 microarray was used to analyze the miRNA expression profiles of 3-MA-treated MCF-7/ADR, untreated MCF-7/ADR and ISL-treated MCF-7/ADR cells. Finally we identified 47 miRNA genes that were differentially expressed (*P*<0.05) among the three groups (Figure 4A). The autophagy activity in the ADR cells was significantly higher than in the MCF-7 cells, and ISL further promoted the autophagy process in the ADR cells; therefore, the miRNA differences among the three groups were expected to follow a pattern of ISL>ADR>3-MA or ISL<ADR<3-MA. We identified 4 miRNAs that followed these patterns; they included miR-210, miR-25, miR-4458 and miR-30b. Using qPCR, we validated miRNA-25 as the target of ISL-triggered autophagy (Figure 4B).

To investigate the effect of miRNA-25 on autophagy in ADR cells, MCF-7/ADR cells were transfected with miR-25 inhibitors or scrambled miRNA as a negative control. Then, autophagy flux was analyzed *via* LC3-II detection. The results revealed that the inhibition of miR-25 led to a significant increase in GFP-LC3-II puncta, indicating that miR-25 may be a novel independent modulator of autophagy (Figure 4C). The miR-25 inhibitors also resulted in decreased ADR cell proliferation and in increased cytotoxicity from epirubicin, indicating that miR-25 inhibition may activate autophagic cell death (Figure 4D). To explore the mechanisms by which miR-25 inhibition induced autophagy, we predicted miR-25 targets using both TargetScan and Microrna.org software. The results showed that the autophagy-related genes ATG14 and ULK1 were involved with the candidate miR-25 targets. We then performed qPCR to validate this possibility. The results showed that after treatment with miR-25 inhibitors, the expression of ULK-1 mRNA in MCF-7/ADR cells was significantly increased, whereas there was little change in the amount of ATG-14 mRNA (Figure 4E). Western blotting results further confirmed that miR-25 inhibition resulted in the increased expression of ULK1, but not of ATG-14 (Figure 4F).

To confirm that the upregulation of ULK1 mRNA was due to a direct decrease in the interaction between miR-25 and its predicted binding site, we cotransfected ADR cells with miR-25 and pMiR luciferase reporter constructs containing 3′ untranslated region (UTR) fragments of ULK1. The relative luciferase activity, assessed 36 h after the transfection of the ULK1 constructs, was reduced by 40.1% in the presence of miR-25 but was upregulated by 35.8% when we transfected miR-25 inhibitors with the ULK1 constructs. More importantly, when we mutated the miR-25 binding sites in the 3′UTR region of ULK1, miR-25 mimics or inhibitors had little effect on the transcriptional luciferase activity, indicating that miR-25 bound directly to the 3′UTR region of ULK1 to affect autophagy (Figure 4G).

### ISL triggers the autophagic cell death of MCF-7/ADR cells primarily by regulating miR-25

**Figure 5:**
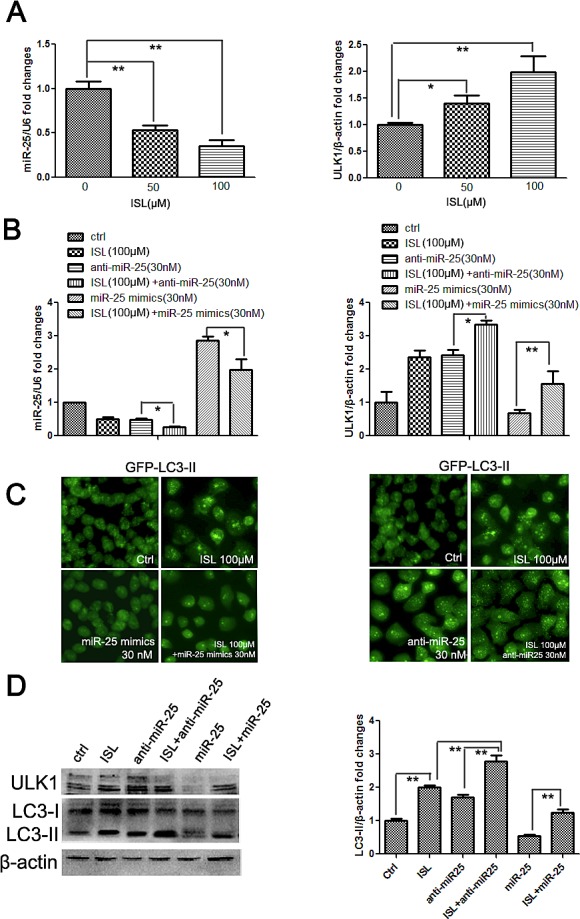
ISL induces the autophagic cell death of MCF-7/ADR cells by inhibiting miR-25 expression (A) qPCR results demonstrated that ISL dose-dependently decreased miR-25 expression and increased the transcription of its target ULK1 after 24 h of treatment (the values represent the means ± SD, n=3, *P<0.05, **P<0.01); (B) the inhibitory effects of ISL on miR-25 expression were exacerbated by the addition of miR-25 inhibitors but were reversed by the addition of miR-25 mimics. Similar effects on ULK1 expression were found, indicating that miR-25 was the primary target of ISL (the values represent the means ± SD, n=3,*P<0.05); (C–D) treatment with miR-25 mimics significantly lowered the LC3-II accumulation induced by ISL treatment at 24 h, whereas miR-25 inhibitors further enhanced the ISL-induced increase in LC3-II expression, indicating that ISL induced autophagy flux primarily by regulating miR-25. These results were further validated using western blotting to reveal the expression levels of ULK1 and LC3-II (the values represent the means ± SD, n=3, **P<0.01).

Based on the clarification of the relationship between miR-25 and autophagy, we sought to validate the critical role of miR-25 in ISL-induced autophagy. We first assessed changes in miR-25 after ISL treatment. The results of qPCR confirmed that ISL dose-dependently inhibited miR-25 expression in ADR cells, which was consistent with the results from the miRNA array. The level of ULK1 mRNA was significantly elevated by ISL, further implying that ULK1 was the direct target of miR-25 (Figure 5A). Interestingly, the downregulation of miR-25 and the upregulation of ULK1 by ISL were reversed after treatment with miR-25 mimics; however, the downregulation of miR-25 and the upregulation of ULK1 were aggravated by miR-25 inhibitors, implying that ISL modulated autophagy flux primarily *via* miR-25 regulation (Figure 5B). To further confirm the pivotal role of miR-25 in mediating ISL-induced autophagy, we performed fluorescence detection of LC3-II, which provided the most direct evidence to support our hypothesis. The results showed that miR-25 mimics dose-dependently inhibited the autophagy induced by ISL, whereas miR-25 inhibitors severely accelerated LC3-II accumulation (Figure 5C). Additionally, western blotting results further revealed that miR-25 mimics inhibited LC3-II and ULK1 expression, whereas the addition of miR-25 inhibitors further increased their levels compared with ISL alone, further confirming that ISL induced autophagy primarily *via* miR-25 inhibition (Figure 5D).

### ISL inhibits miR-25 and triggers autophagy in a drug-resistant breast cancer xenograft

**Figure 6:**
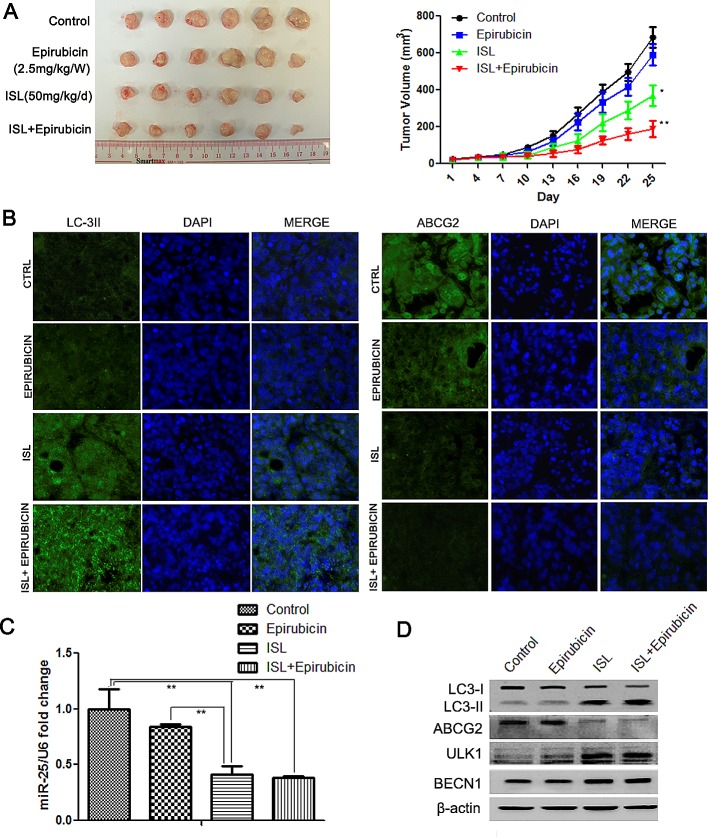
ISL reverses breast cancer drug resistance by inducing autophagy miR-25 downregulation (A) ISL synergistically interacted with epirubicin to inhibit the growth of drug-resistant breast cancer xenografts (the values represent the means ± SD, n=6, *P<0.05, **P<0.01 vs. epirubicin group); (B) immunofluorescence results showed that ISL induced the upregulation of LC3-II and the downregulation of ABCG2 in breast cancer tissues; (C) qPCR results confirmed that ISL inhibited miR-25 expression *in vivo* (the values represent the means ± SD, n=3,**P<0.01); (D) western blotting validated that ISL inhibited ABCG2 expression and activated autophagy markers *in vivo*, including the upregulation of LC3-II, ULK1 and BECN1.

To investigate whether ISL also chemosensitized breast cancer *via* autophagy induction *in vivo*, we constructed a drug-resistant breast cancer xenograft by injecting MCF-7/ADR cells into the mammary pads of NOD/SCID mice. The mice were then treated with epirubicin or ISL. The results showed that epirubicin had little effect in limiting cancer growth, whereas ISL alone significantly inhibited breast cancer volume. The inhibitory effects were the highest when ISL and epirubicin were applied together to treat the drug-resistant breast cancer (Figure 6A). The *in vivo* results were in consistent with our *in vitro* findings. Immunofluorescence detection revealed that ISL increased the LC3-II expression in breast cancer tissue but decreased the expression of ABCG2 (Figure 6B), indicating that the chemosensitizing effects of ISL *in vivo* were closely connected to autophagy induction. To validate whether the *in vivo* activation of autophagy was induced by miR-25 downregulation, we measured the expression of miR-25 in drug-treated groups. The results showed that ISL with or without epirubicin significantly inhibited miR-25 expression in the tumor tissue (Figure 6C); this was accompanied by increases in the expression of LC3-II, ULK1 and BECN1 as well as a decrease in the expression of ABCG2 (Figure 6D). Taken together, our data demonstrated that ISL induced the autophagic cell death of drug-resistant breast cancer cells both *in vitro* and *in vivo* and that miR-25 functioned as the primary autophagy-related modulator by targeting ULK1.

## DISCUSSION

In the past two decades, a number of studies have demonstrated that compounds with low toxicity, such as quercetin, epigallocatechin-3-gallate and curcumin, were excellent in reversing drug resistance in various cancer types [36-38]. Although the molecular mechanisms of these compounds were shown to involve multiple signaling pathways, scarce attention was paid to the roles of autophagy-related pathways in regulating chemoresistance. Autophagy is now emerging as a crucial player in the response to metabolic and therapeutic stresses by attempting to maintain cellular homeostasis by eliminating excessive or unnecessary proteins and damaged organelles *via* lysosomal degradation. Pharmacological modulation of autophagy has become a promising strategy to overcome cancer drug resistance [39]. Herein, we revealed that the natural chalcone-type compound ISL aggravated autophagy in drug-resistant breast cancer cells, which was accompanied by a G2/M checkpoint arrest and the downregulation of ABCG2 expression. Apoptosis assays indicated that ISL treatment of MCF-7/ADR cells led to limited apoptosis but decreased expression of Bcl-2. Bcl-2 is well documented to inhibit autophagy *via* a physical interaction with BECN1, an essential molecule in autophagy induction [32]; our results provided indirect evidence supporting the autophagy-induced effects of ISL. The addition of the autophagy inhibitors 3-MA and CQ reduced the cell death caused by ISL treatment to some degree, further demonstrating that autophagic cell death could be the primary mechanism of the chemosensitizing effects of ISL. Given the significant role of ABCG2 in mediating drug resistance and our observation that ISL inhibited ABCG2 expression in ADR cells, we examined whether the inhibition of ABCG2 by ISL was caused by autophagy induction. Interestingly, we found that in ADR cells, the autophagy induction occurred significantly sooner after ISL treatment than the downregulation of ABCG2. The autophagy inhibitors 3-MA and CQ restored the ABCG2 expression inhibited by ISL, indicating that the decrease in ABCG2 was primarily caused by enhanced autophagy-lysosome degradation.

Recent findings have suggested that dysregulated miRNAs were closely connected to autophagy modulation and cancer drug resistance. Zhu *et al.* first reported that miR-30a functioned in autophagy and cancer by targeting BECN1 [40]. Emerging evidence further supports the functional significance of miR-30a-mediated autophagy in chemosensitizing cancer cells to anticancer drugs [41, 42]. Moreover, a number of miRNAs, such as miR-130a, miR-101, miR-375 and miR-23b have also been reported to participate in autophagy modulation [20]. miRNAs possess the ability to simultaneously target multiple genes and pathways, which is a potential advantage for drug design and pharmacological studies. To study whether miRNAs were also involved in ISL-mediated autophagy, an miRNA array was used to identify the potential target of ISL. A comparison of the different levels of expression of miRNAs in MCF-7, MCF-7/ADR and ISL-treated cells revealed that the miR-25 level was gradually inhibited with increasing autophagic activity. To further validate the significant role of miR-25 in regulating autophagy, we transfected cells with miR-25 mimics or inhibitors. The results revealed that miR-25 inhibition led to increased autophagy and cell death, indicating that miR-25 was an independent factor contributing to autophagy modulation. Previously, the understanding of the roles of miR-25 in cancer biology and autophagy induction was limited. The present study was the first to identify miR-25 as an autophagy modulator with a role in drug resistance in breast cancer.

To further elucidate the molecular mechanism of miR-25-mediated autophagy, we sought to identify its putative target genes. Using software prediction and a luciferase reporter assay, we identified the autophagy-related gene ULK1 as a direct target of miR-25 and confirmed the upregulation of ULK1 expression in ADR cells after the transfection of miR-25 inhibitors. In mammalian cells, autophagy induction is initiated by the ULK complex, which is composed of ULK1 or ULK2, ATG13, FIP200 and ATG101. Under autophagy-inducing conditions, this complex immediately senses stress signals from upstream molecules such as mTORC1 and AMPK, resulting in the dephosphorylation of specific residues on ULK1/2 or ATG13 to activate phagophore formation [43, 44]. Although numerous studies have demonstrated that ULK1 knockdown was sufficient to block starvation-induced autophagy in multiple cell types [45, 46], the mechanism underlying ULK1-mediated autophagy remained largely unknown. A recent study found that activated ULK1 directly phosphorylated BECN1 on Ser14, enhancing the activity of Atg14L-containing VPS34 complexes and ultimately promoting autophagy induction and maturation [47]. In the present study, we found that after treatment with miR-25 inhibitors, the downstream autophagic signaling molecules BECN1 and LC3-II were both upregulated, further implying that miR-25 functioned as a modulator in the early phase of autophagy induction. Previously, only miR-885-3p had been reported to regulate early autophagy by targeting ULK2 [48]; our findings clearly expand the knowledge concerning miRNA regulators in this phase.

To further validate the central role of miR-25 in ISL-induced autophagy, we measured changes in the levels of miR-25 and its target gene ULK1 following ISL treatment. The results confirmed that ISL dose-dependently inhibited miR-25 expression, thus promoting ULK1 activation. Additionally, the addition of miR-25 mimics effectively abrogated the autophagy effects of ISL and therefore restored cell proliferation, implying that miR-25 is the primary target of ISL in autophagy induction. Over the last decade, accumulating evidence has indicated that ISL functions a potent anticancer agent with chemopreventive and chemotherapeutic potential by interrupting multiple survival signaling, such as COX-2, PI3K/Akt and NF-κB. [49-52]. However, the precise mode of action of ISL has not been completely understood, hindering its incorporation into mainstream clinical treatment regimens. The discovery of miRNAs provided a timely and powerful tool to elucidate the multitarget nature of natural compounds. To our knowledge, this is the first report to identify an miRNA target of ISL involved in chemosensitizing breast cancer by autophagy induction. To determine whether ISL treatment could result in similar chemosensitizing effects *in vivo*, we constructed a breast cancer xenograft with MCF-7/ADR cells. The results revealed that ISL exerted synergistic effects with epirubicin and induced autophagy *in vivo*, indicated by increased LC3-II and decreased ABCG2 expression in tumor tissues. The level of miR-25 in ISL-treated tumors was also downregulated, which was accompanied by an increase in the expression of ULK1, which was consistent with our *in vitro* findings. The results suggest that ISL is a physiological inducer of autophagy *in vivo*.

In conclusion, our results suggest that ISL has potential for development as a chemosensitizing agent for breast cancer due to its ability to inhibit miR-25 expression, which in turn leads to the upregulation of its target gene ULK1 and the induction of autophagic cell death, ultimately resulting in accelerated ABCG2 degradation *via* the lysosome pathway. Our investigation not only revealed a novel mechanism of ISL in inducing autophagic cell death to overcome drug resistance but also identified miR-25 as a novel target for regulating autophagy and chemosensitivity. However, future studies are needed to determine the clinical implications of miR-25 as a prognostic factor in breast cancer or other cancer types. Moreover, the role of miR-25 in regulating autophagy *via* targeting ULK1 remains to be validated in other cancer models.

## MATERIALS AND METHODS

### Chemicals, reagents and antibodies

The chemotherapeutic drug epirubicin was purchased from Sigma (St. Louis, MO). ISL, with a purity of more than 97%, was purchased from Alpha Aesar (MA, USA). A stock solution of ISL was prepared in dimethyl sulfoxide (DMSO) and stored at -20°C. ISL was diluted in the culture medium to obtain the desired concentration. Propidium iodide (PI), 3-(4,5-dimethylthiazol-2-yl)-2,5-diphenyltetrazolium bromide (MTT), 4,6-diamino-2-phenylindole (DAPI), AO and 3-MA were obtained from Sigma. CQ was purchased from InvivoGen (San Diego, CA). Antibodies against LC3, PARP, Bax, Bcl-2, caspase 3, ULK1, ATG14 and β-actin as well as the Alexa Fluor 488 conjugate were purchased from Cell Signaling Technology (Danvers, MA). Antibodies against ABCG2, p-mTOR and BECN1 as well as fluorescently labeled secondary antibodies were purchased from Santa Cruz Biotechnology (Dallas, TX). The X-tremeGENE siRNA Transfection Reagent was purchased from Roche Diagnostics (Indianapolis, IN).

### Cell lines and culture

The human breast cancer cell line MCF-7 and the normal human mammary epithelial cell line MCF-10A were obtained from the American Type Culture Collection. MCF-7/ADR cells were derived by treating MCF-7 cells with gradually increasing epirubicin for six months. MCF-7 and MCF-7/ADR cells were cultivated in RPMI-1640 supplemented with 10% fetal bovine serum (FBS) and 1% penicillin and streptomycin at 37°C in a humidified incubator with 5% CO_2_. The MCF-10A cells were maintained in Keratinocyte-SEM medium (Gibco, Carlsbad, CA) supplemented with 30 μg/ml bovine pituitary extract, 0.2 ng/ml rEGF and 1% penicillin and streptomycin (Gibco).

### Cell proliferation analysis and colony formation assay

The effects of epirubicin or ISL on cell proliferation were studied using MTT as previously described [52]. Briefly, cells were seeded onto a 96-well plate at a density of 4×10^3^ cells/well. After serum starvation, different concentrations of epirubicin or ISL were added to the wells, with 6 repeats for each concentration. Cell viability was assessed after 24 h using MTT according to the manufacturer's instructions. Independent experiments were performed in triplicate. To evaluate the long-term inhibitory effects of ISL on cell proliferation, a colony formation assay was performed. MCF-7/ADR cells were seeded onto 6-well plates at a density of 1,000 cells/well. After cell attachment, epirubicin or ISL (alone or in combination) was added to the wells for 4 h; the cells were then cultured with fresh medium. After 2 weeks, the formed colonies were fixed with 4% paraformaldehyde and stained with coomassie blue. The colonies were then photographed and counted under a microscope.

### Flow cytometry analysis

For the cell cycle analysis, MCF-7/ADR cells (3×10^5^/well) were synchronized at G1 phase by serum deprivation and were then treated with ISL. The cells were harvested after 24 h. PI-stained single-cell suspensions were analyzed using a FACSCalibur (BD Bioscience), and the CellQuest and ModFit programs were used for data analysis. For the apoptosis assay, cells were seeded onto 6-well plates at a density of 5×10^5^/well. After ISL treatment for 24 h, the cells were stained with Annexin V-FITC (Bender MedSystems, Vienna, Austria) for 15 min at room temperature. The apoptotic index was determined using FACSCalibur with FlowJo software. For the drug efflux assay, breast cancer cells were incubated with epirubicin (1 μg/ml) for 60 min at 37°C. After washing, the cells were released in drug-free medium for 90 min and subjected to flow cytometry.

### miRNA 3.0 array analysis

Total RNA was extracted from the 3-MA-treated MCF-7/ADR, untreated MCF-7/ADR and ISL-treated MCF-7/ADR cells using the mirVana miRNA Isolation Kit (Ambion, Austin, TX). A microarray chip analysis was performed using the Affymetrix miRNA 3.0 Array (Agilent Technologies, Palo Alto, CA), which is composed of 19,913 probe sets for miRNAs registered in the Sanger miRBase miRNA database, version 20 (June 2013). Prior to the microarray analysis, the RNA quality was confirmed using the RNA Integrity Number algorithm of the Agilent 2100 Bioanalyzer (Agilent Technologies) at the University of Hong Kong. All of the samples had RNA Integrity Numbers greater than 7.0. Partek Genomics Suite 6.6 software was used to identify differences in miRNA expression between groups with fold changes >|2|. The TargetScan and Microrna.org programs were then applied to predict potential protein targets and binding sites of the selected miRNAs.

### miRNA precursor molecules, isolation and qPCR analysis

All the synthetic miRNA mimics and inhibitors as well as scrambled miRNAs were purchased from Invitrogen. For the miRNA isolation and qPCR analysis, total RNA was extracted from the cells using the mirVana miRNA Isolation Kit. The miRcute miRNA First-strand cDNA Synthesis Kit (Tiangen, Beijing, China) and qPCR Detection Kit (Tiangen) were used to measure the expression levels of miR-25, -30b, -4458 and -210 before and after drug treatment; human U6 snRNA served as an internal control. For mRNA quantification, total RNA was isolated with TRIzol reagent, and reverse transcription was performed using a First-strand cDNA Synthesis Kit (Roche, Mannheim, Germany) according to the manufacturer's instructions. Real-time PCR was performed using a SYBR Green kit (Roche) on the Roche LightCycler 480 platform. The primers for miRNAs and mRNAs are described in Supplementary Table 1. The Ct value was measured during the exponential amplification phase. The relative expression level (defined as the fold change) of each target gene was determined using the 2^−ΔΔCt^ method and was normalized to the fold change.

### Western blotting

Western blotting was performed as previously described [15]. Quantified protein lysates (20 μg) were resolved in an SDS-PAGE gel and were transferred onto a PVDF membrane. The membrane was then blocked in 5% bovine serum albumin (BSA) for 2 h, washed with TBS and incubated with the primary antibody overnight at 4°C. After incubation with the secondary antibody for 2 h at room temperature, the immunoreaction was visualized using the ECL Advance reagent (GE Healthcare) and was quantified using Quantity One software.

### Generation of cell lines stably expressing GFP-LC3-II

The pSelect-GFP-hLC3 plasmid was purchased from InvivoGen and was transfected into MCF-7/ADR cells using Lipofectamine 2000 (Invitrogen). After 24 h, the transfected cells were passaged and selected for 2 weeks with 200 μg/ml Zeocin (InvivoGen). Pooled populations of positive cells were obtained 2 weeks after drug selection without subcloning and used in *in vitro* experiments.

### Luciferase reporter gene assay

The 3′UTR fragments of ULK1 containing the miRNA target sites were PCR-amplified from an MCF-7/ADR cDNA library using FastStart Taq DNA polymerase (Roche, Basel, Switzerland). The PCR products were cloned into the HindIII and XhoI restriction sites of the pMIR-REPORT luciferase plasmid (Ambion). The mutant 3′UTR segments of the human ULK1 mRNA, which harbored mutations in the complementary site for the seed region of miR-25, were generated by performing site-directed mutagenesis of the wild-type segment. The primers used are listed in Supplementary Table 1. For the reporter assay, 1×10^5^ MCF-7/ADR cells were cotransfected with 5 ng of 3′UTR reporter plasmid, 50 ng of the pRL-TK *Renilla* luciferase reporter vector (Promega, Madison, WI) and 30 nM of miR-25 mimics or inhibitors (Invitrogen) using the X-tremeGENE siRNA Transfection Reagent. The transfected cells were lysed 36 h after transfection, and the luciferase activity was assayed with the Dual-Luciferase Reporter System (Promega).

### Immunofluorescence analysis and AVO imaging

The expression of ABCG2 in ADR cells before and after ISL treatment was observed using immunofluorescence. Cells were cultured on glass coverslips with or without ISL treatment. After three washes with PBS, the cells were fixed in 4% paraformaldehyde and permeabilized with 0.05% Triton X-100. The fixed cells were then blocked with 5% goat serum and incubated with primary antibodies overnight. After washing, the cells were incubated with Alexa Fluor-conjugated secondary antibodies and counterstained with DAPI. For AVO observation, drug-treated or untreated cells were incubated with 1 μg/ml AO in PBS at 37°C for 15 min. After three washes with PBS, the cells were visualized using fluorescence microscopy.

### Breast cancer xenografts

All the animal studies involving animal experiments were reviewed and approved by the University of Hong Kong's Committee for the Ethical Review of Research. MCF-7/ADR cells were resuspended in PBS at 5×10^6^ cells/100 μl and were injected into the fourth mammary gland fat pad of 4- to 5-week-old female NOD/SCID mice. Each animal received subcutaneous injections of 1.5 mg of 17β-estradiol (Sigma) at three-day intervals to support cancer growth. Tumors were measured with a caliper at 3-day intervals, and the tumor volume was calculated using the following formula: volume (mm^3^)=width^2^×length/2. After the tumor size reached approximately 5 mm×5 mm, epirubicin and ISL were administered by intraperitoneal injection at 2.5 mg/kg/W and 50 mg/kg/d. The tumor tissues were removed at the end of the experiment and subjected to histological examination or western blotting. For immunofluorescence detection, tumor samples were rinsed in PBS and fixed in 4% paraformaldehyde. The samples were then dehydrated in an ethanol gradient, paraffin-embedded and sectioned (4 μm) for analysis. The following procedures were the same as described above.

### Statistical analysis

The data were expressed as means ± standard deviations (SD). A two-tailed Student's t-test was used to determine the significance of differences between groups. Results with *P* values <0.05 were considered statistically significant.

## SUPPLEMENTARY MATERIAL TABLE


