# Supplementary material for: MicroRNA-25 regulates chemoresistance-associated autophagy in breast cancer cells, a process modulated by the natural autophagy inducer isoliquiritigenin
Source: Oncotarget. 2014 Jul 9;5(16):7013–26. doi: 10.18632/oncotarget.2192 (PMC4196180; doi:10.18632/oncotarget.2192)
Supplement: Supplementary file 1 [file oncotarget-05-7013-s001.doc]

| **Primers for QPCR** | |
| --- | --- |
| miR-210-3P | 5’-GTGGGGAGAGGTAGGTGTGGAAGAA-3’ |
| miR-25-3P | 5’-TGTTTTTTCATTGCACTTGTCTCG-3’ |
| miR-30b-5P | 5’-GTGCTGTGTGCTGTCAACATCCTACAC-3’ |
| miR-4458-5P | 5’-GTGGGGAGAGGTAGGTGTGGAAGAA-3’ |
| U6-F | 5’-CTCGCTTCGGCAGCACA-3’ |
| U6-R | 5’-AACGCTTCACGAATTTGCGT-3’ |
| ULK1-F | 5’-gcttagggcaggtcagca-3’ |
| ULK1-R | 5’-tgggtatctcggagggaga-3’ |
| ATG-14-F | 5’-tggggactactctgcctactaca-3’ |
| ATG-14-R | 5’-gggttactctgctccatgtca-3’ |
| β-actin-F | 5’-CCAACCGCGAGAAGATGA-3’ |
| β-actin-R | 5’-CCAGAGGCGTACAGGGATAG-3’ |
| **Primers for ULK1 3’UTR cloning** | |
| 3’UTR-F | 5’-CCCAAGCCTCCTTTCTGGCCTGGC-3’ |
| 3’UTR-R | 5’-CCGCTCGAGGGTTTATTTCCTTACG-3’ |
| Mutant 3’UTR-F | 5’-cacacacgtgtgtttttcttgaaccgacttgaaatattgcc-3’ |
| Mutant 3’UTR-R | 5’-ggcaatatttcaagtcggttcaagaaaaacacacgtgtgtg-3’ |

**Supplementary Table 1**
